# The guard cell metabolome: functions in stomatal movement and global food security

**DOI:** 10.3389/fpls.2015.00334

**Published:** 2015-05-19

**Authors:** Biswapriya B. Misra, Biswa R. Acharya, David Granot, Sarah M. Assmann, Sixue Chen

**Affiliations:** ^1^Department of Biology, Genetics Institute, Plant Molecular and Cellular Biology Program, University of Florida, Gainesville, FL, USA; ^2^Department of Biology, Pennsylvania State University, PA, USA; ^3^Department of Vegetable Research, Institute of Plant Sciences, Agricultural Research Organization, Bet-Dagan, Israel; ^4^Interdisciplinary Center for Biotechnology Research, University of Florida, Gainesville, FL, USA

**Keywords:** stomata, primary metabolites, abscisic acid, phytohormones, lipids, specialized metabolites, food security

## Abstract

Guard cells represent a unique single cell-type system for the study of cellular responses to abiotic and biotic perturbations that affect stomatal movement. Decades of effort through both classical physiological and functional genomics approaches have generated an enormous amount of information on the roles of individual metabolites in stomatal guard cell function and physiology. Recent application of metabolomics methods has produced a substantial amount of new information on metabolome control of stomatal movement. In conjunction with other “omics” approaches, the knowledge-base is growing to reach a systems-level description of this single cell-type. Here we summarize current knowledge of the guard cell metabolome and highlight critical metabolites that bear significant impact on future engineering and breeding efforts to generate plants/crops that are resistant to environmental challenges and produce high yield and quality products for food and energy security.

## Introduction

Guard cells as a unique plant single cell-type perform many functions essential to plant growth and survival. Each pair of guard cells and the regulated pore they enclose, known as a stoma or stomate, provides a conduit for atmospheric photosynthetic gas exchange (CO_2_ uptake and O_2_ release) and transpirational release of water (H_2_O) in terrestrial plants, in addition to defense against pathogenic invasion. Stomatal opening and closing, in which the guard cells actively increase and decrease their volume via turgor changes to regulate the pore size in response to environmental stimuli, are vital processes in maintaining the balance of H_2_O loss and CO_2_ fixation. While drought stress induces stomatal closure, pathogens exploit stomatal opening to facilitate entry into the leaf (Zeng and He, 2010). Abscisic acid (ABA), CO_2_ and blue light mediated stomatal movements have generated tremendous interest in their signaling mechanisms. Each pathway/network has unique components such as distinct receptors and early signaling elements. They also have common components, for example, actual stomatal movement is caused by water influx/efflux mostly driven by solute fluxes through plasma membrane anion channels and K^+^ channels. When the concentrations of solutes decrease in guard cells in the cases of ABA and elevated CO_2_, water potential increases in the cells and water flows out, causing a decrease of turgor pressure and closure of the pores. Blue light activates H^+^-ATPases and resultant membrane hyperpolarization drives K^+^ influx, leading to decreased water potential, increased turgor pressure, and stomatal opening. Please refer to excellent articles published over the years on these signaling mechanisms (e.g., Zeiger and Zhu, 1998; Schroeder et al., 2001; Yu and Assmann, 2014; Zhang et al., 2014; Tian et al., 2015). Although stomatal movements in response to ABA, CO_2_, and blue light are well studied, the metabolome of the guard cell is far from catalogued. Endeavors in metabolomic approaches have led to deeper understanding of the biology inherent to several specialized important single-cell types including guard cells (Misra et al., 2014). Recent efforts to comprehend the guard cell metabolome (Jin et al., 2013) and systems biology approaches to identify the critical regulators in stomatal movement (Sun et al., 2014) have provided interesting leads into the intricate regulation of stomatal movement in response to environmental stimuli. Ongoing systems biology approaches, combining modeling and high-throughput experiments, will help to elucidate the mechanisms underlying stomatal control and unravel targets for modulation of stomatal responses to environment (Medeiros et al., 2015).

**FIGURE 1:**
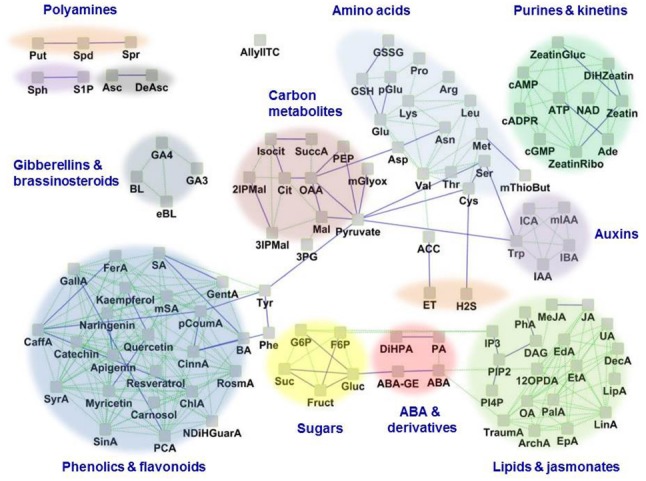
**The guard cell metabolome.** Based on available literature, 109 known metabolites in guard cells are represented as a network based on their structural and biochemical relationships. Solid blue lines represent KEGG pathway-based biochemical relatedness and green dotted lines represent the Tanimoto structural-index based relatedness, which were inferred using the MetaMapR tool (http//dgrapov.github.io/MetaMapR/) and were visualized using Cytoscape (http://www.cytoscape.org/). The clusters of metabolites are highlighted based on metabolic categorization. Abbreviations of metabolites are as follows: ABA, Abscisic acid; ABA-GE, Abscisic acid glucose ester; ACC, 1-Amino cyclopropane-1-carboxylic acid; Ade, Adenine; ArchA, Arachidonic acid; Arg, L-Arginine; Asc, Ascorbic acid; Asn, L-Asparagine; Asp, L-Aspartic acid; ATP, Adenosine triphosphate; AllylITC, Allylisothiocyanate; BA, Benzoic acid; BL, Brassinolide; cADPR, Cyclic adenosine diphosphate ribose; CaffA, Caffeic acid; cAMP, Cyclic adenosine monophosphate; ChlA, Chlorogenic acid; CinnA, trans-Cinnamic acid; Cit, Citric acid; Cys, L-Cysteine; DAG, 1,2-Diacylglycerol; DAsc, Dehydroascorbate; DecA, Decanoic acid; DiHPA, Dihydrophaseic acid; eBL, Epibrassinolide; EdA, Eicosadienoic acid; EpA, Eicosapentaenoic acid; ET, Ethylene; EtA, Eicosatrienoic acid; FerA, Ferulic acid; F6P, Fructose-6-phosphate; Fruct, Fructose; GallA, Gallic acid; GA3, Gibberellic acid A3; GA4, Gibberellic acid A4; GentA, Gentisic acid; Glu, L-Glutamic acid; Gluc, Glucose; G6P, Glucose-6-phosphate; GSH, Glutathione (reduced); GSSG, Glutathione (oxidized); His, L-Histidine; H_2_S, Hydrogen sulfide; IAA, Indole-3-acetic acid; IBA, Indole-3-butyric acid; ICA, Indole-3-carboxylic acid; I3P, Inositol-1, 4, 5-trisphosphate; 2IPMal, 2-Isopropylmalic acid; 3IPMal, 3-Isopropylmalic acid; Isocit, DL-Isocitric acid; JA, Jasmonic acid; Leu, L-Leucine; LinA, Linoleic acid; LipA, α-Lipoic acid; Lys, L-Lysine; Mal, L-Malic acid; MeJA, Methyl jasmonate; Met, L-Methionine; mGlyox, Methylglyoxal; mIAA, Methyl indole-3-acetate; mSA, Methyl salicylate; mThioBut, α-keto-γ-(methyl-thio) Butyric acid; mSA, Methyl salicylic acid; NAD, Nicotinamide adenine dinucleotide; NDiHGuarA, Nor dihydro guaiaretic acid; OA, Oleic acid; OAA, Oxalo acetic acid; OlAEE, Oleic acid ethyl ester; 12OPDA, 12-oxophytodienoic acid; cGMP, Guanosine-3′,5′-cyclic monophosphate; PA, Phaseic acid; PalA, Palmitic acid; PCA, Protocatechuic acid; pCoumA, p-Coumaric acid; PEP, Phosphoenolpyruvate; 3PG, 3-Phosphoglycerate; PhA, Phosphatidic acid; pGlu, L-Pyroglutamic acid; Phe, L-Phenylalanine; Pinitol, D-Pinitol; PI3P, Phosphatidylinositol-3-phosphate; PI4P, Phosphatidylinositol-4-phosphate; PIP2, Phosphatidylinositol-4, 5-bisphosphate; Pro, L-Proline; Put, Putrescine; RosmA, Rosmarinic acid; SA, Salicylic acid; Ser, L-Serine; SinA, Sinapinic acid; S1P, Sphingosine-1-phosphate; Sph, D-erythro-Sphingosine; Spd, Spermidine; Spr, Spermine; Suc, Sucrose; SuccA, Succinic acid; SyrA, Syringic acid; Thr, L-Threonine; TraumA, Traumatic acid; Trp, L-Tryptophan; Tyr, L-Tyrosine; UA, Undecanoic acid; Val, L-Valine; Zeatin, trans-Zeatin; ZeatinGluc, trans-Zeatin glucoside; ZeatinRibo, trans-Zeatin riboside.

Many factors pose immense challenges to global food and bioenergy security, including population growth, climate, and environmental changes coupled to land degradation and changes in hydrological resources, essential ecosystem services, and agricultural production systems. Urgent efforts are needed to enhance the resilience of crops to the adverse effects of climate change. Stomata are highly responsive to hormonal and environmental cues, including those associated with climate change: water availability, temperature, and CO_2_ concentrations. Thus, understanding the basic biology, the concealed information content, and the connection to functional output of guard cells through multiple -omics approaches such as transcriptomics (Wang et al., 2011), proteomics (Zhao et al., 2008; Zhu et al., 2014) and metabolomics (Jin et al., 2013) is highly relevant to the goal of improving crop productivity and yield in ever changing climatic regimens. Here we briefly review collective efforts to unravel the functional guard cell metabolome (Figure 1), to discover metabolites of convergence and divergence among various environmental cues, to examine the molecular mechanisms of guard cell metabolic regulation, and ultimately to highlight the potential of guard cell biology in harnessing possible solutions for global food and bioenergy security.

## Primary Metabolites of Carbon Metabolism in Regulatory Roles of Stomatal Function

Early hypotheses regarding guard cell osmoregulation suggested that sugar generated from starch degradation at dawn is the primary osmolyte that opens stomata (Lloyd, 1908). Upon the discovery that potassium (K^+^) ions, with chloride (Cl^–^) and malate^2–^ as counter anions, are osmolytes that open stomata, a role for sugar in guard cell osmoregulation and stomatal movement was abandoned for several decades (Imamura, 1943; Yamashita, 1952; Fischer, 1968; Humble and Raschke, 1971; Allaway, 1973; Outlaw and Lowry, 1977; Asai et al., 2000). A later study reporting that blue and red (photosynthetic) light can open stomata and are followed by sucrose accumulation in guard cells revived the hypothesis of sucrose as an osmolyte that opens stomata (Talbott and Zeiger, 1993). A correlation between the decline of K^+^ content in guard cells in the middle of the day concomitantly with an increase in sucrose content further suggested that sucrose is an osmolyte that replaces K^+^ and maintains stomatal opening (Amodeo et al., 1996; Talbott and Zeiger, 1996).

The origin of sucrose in guard cells is not yet clear. Potentially, sucrose could be obtained from guard cell starch degradation, guard cell photosynthesis, or import from mesophyll cells (Gotow et al., 1988; Talbott and Zeiger, 1988; Lawson et al., 2014). It is generally accepted though, that the contribution of sucrose produced from guard cell photosynthesis to the osmotic requirement for stomatal opening is minimal and that most of the sugar or the organic compounds from which sugar can be synthesized is obtained from the mesophyll cells (Outlaw, 1989; Reckmann et al., 1990). When exported out of the mesophyll cells for phloem loading, some sucrose accumulates in the guard cell apoplast (Lu et al., 1995, 1997; Outlaw and De Vlieghere-He, 2001). As a result, the concentration of sucrose in the guard cell apoplast increases as photosynthesis proceeds. This sucrose may be imported by guard cells and contribute to guard cell osmolarity and stomatal opening. But it also has been proposed that as sucrose accumulates in the apoplast, its osmotic effect drives water efflux from guard cells, resulting in a decrease in stomatal apertures in a mechanism that thus inversely coordinates photosynthesis and transpiration rates (Lu et al., 1997; Outlaw and De Vlieghere-He, 2001).

Apoplastic sucrose may enter the guard cells either via sucrose transporters, or via guard cell hexose transporters following sucrose cleavage by apoplastic invertase to yield the hexoses glucose and fructose (Stadler et al., 2003; Weise et al., 2008; Bates et al., 2012). Regardless of its origin, mesophyll or guard cells, sucrose must be cleaved to be metabolized, and the hexoses obtained from sucrose cleavage, glucose and fructose, must be phosphorylated by intracellular hexose phosphorylating enzymes, hexokinases (HXK) and fructokinases (FRK; Dennis and Blakeley, 2000). Glucose can be phosphorylated only by HXK, an enzyme demonstrated to exist in guard cells and to participate in sugar sensing (Moore et al., 2003; Rolland et al., 2006; Granot, 2008). A recent study has shown that sugars such as sucrose, glucose, and fructose, do not exert an apoplastic osmotic effect on guard cells, but rather are sensed within guard cells by HXK to stimulate stomatal closure, thus coordinating photosynthesis and sugar levels with transpiration (Kelly et al., 2013).

The phosphorylated hexoses (hexose-P) within guard cells may be converted to starch or enter glycolysis and the tricarboxylic acid (TCA) cycle to yield energy (ATP) and various metabolites including pyruvate and malate that regulate stomatal movement (Allaway, 1973; Pearson, 1973; Outlaw and Lowry, 1977). Glycolysis is a central metabolic pathway for cellular respiration and generation of energy in the form of ATP. In the glycolytic pathway, 2,3-biphosphoglycerate-independent phosphoglycerate mutase (iPGAM) catalyzes the interconversion of 3-phosphoglycerate to 2-phosphoglycerate. *Arabidopsis thaliana* double mutants of *iPGAM* genes show hyposensitivity in blue light, ABA, and low CO_2_ regulated stomatal movements, confirming a role of glycolysis in guard cell function (Zhao and Assmann, 2011). ABA inhibition of stomatal opening in *Commelina benghalensis* is reversed by exogenous ATP and pyruvate (Raghavendra et al., 1976), suggesting a role of pyruvate in negative regulation of ABA signaling (Yu and Assmann, 2014). Recently, it was established that a putative mitochondrial pyruvate importer, NRGA1, negatively regulates ABA inhibition of K^+^ inward channels, ABA activation of slow anion channels and drought tolerance in *A. thaliana* (Li et al., 2014). Altogether, these findings suggest that accumulation of pyruvate in mitochondria would oppose stomatal closure.

Malate, an osmolyte that contributes to stomatal opening, can be generated from hexoses and phosphorylated hexoses obtained from guard cell starch degradation or from triose-phosphates produced in guard cell chloroplasts and exported to the cytoplasm where triose-P metabolism yields malate among other metabolites. ABA-stimulated stomatal closure is accompanied by malate disposal through release, gluconeogenesis, or consumption in the TCA cycle, supporting the role of malate as an osmolyte that opens stomata (Dittrich and Raschke, 1977). In the guard cell cytosol, malate can be metabolized into oxaloacetate (OAA) by malate dehydrogenase. Subsequently, phosphoenolpyruvate carboxykinase (PEPCK) can catalyze the production of PEP from OAA that in turn would enter into gluconeogenesis. An isoform of PEPCK, PCK1, is expressed in *A. thaliana* guard cells according to three experimental approaches: *PCK1* gene promoter analysis and analyses of the proteome, and transcriptome of guard cell protoplasts (Leonhardt et al., 2004; Penfield et al., 2012; and Zhao et al., 2008). Loss-of-function *PCK1* plants (*pck1-2*) show hyposensitivity in response to dark-induced (but not ABA-induced) stomatal closure, indicating the importance of malate metabolism for some stomatal responses (Penfield et al., 2012). Malate produced in photosynthetic tissues may also arrive at and enter the guard cells through malate transporters (Lee et al., 2008). Mesophyll-produced malate also coordinates stomatal behavior with mesophyll photosynthesis, as increasing apoplastic malate activates anion channels that reduce stomatal aperture (Hedrich and Marten, 1993; Fernie and Martinoia, 2009; Araujo et al., 2011). In addition, methylglyoxal, an oxygenated short aldehydic glycolytic intermediate, can induce stomatal closure in *A. thaliana* accompanied by extracellular reactive oxygen species (ROS) production mediated by SHAM-sensitive peroxidases, intracellular ROS accumulation, and suppression of free cytosolic (Ca^2+^) oscillations (Hoque et al., 2012). These results indicate a strong interconnectivity between central carbon metabolism and ABA signaling in guard cells.

## Reactive Oxygen Species Related Metabolites in Guard Cell Signaling

Reactive oxygen species and nitric oxide (NO) are central components of the signaling network regulating stomatal movement in response to ABA, jasmonic acid (JA), darkness, UV, pathogen, and high CO_2_ concentrations (Zhang et al., 2001; Desikan et al., 2004, 2006; Zhu et al., 2012; Akter et al., 2013; He et al., 2013; Joudoi et al., 2013; Ou et al., 2014). Upon application of NO-releasing compounds, NO induces dose-dependent stomatal closure. In contrast, NO has also been implicated as a key component in negative feedback regulation of ABA guard cell signaling through S-nitrosylation of OST1 at cysteine 137 and subsequent inactivation of kinase activity that in turn blocks the positive regulatory role of OST1 in ABA signaling (Wang et al., 2015). NO-mediated negative feedback regulation may prevent complete stomatal closure, allowing some basal level of CO_2_ uptake and photosynthesis. Hydrogen peroxide (H_2_O_2_) may also elicit stomatal movement in a similar manner through redox modification of guard cell signaling components. However, experimental data are lacking for this hypothesis. In addition, ascorbic acid (Asc) and glutathione (GSH) are critical in maintaining cellular ROS levels and redox homeostasis (Noctor and Foyer, 1998). Asc is a key antioxidant that scavenges ROS including H_2_O_2_. Dehydroascorbate reductase (DHAR) is the key regulatory enzyme that catalyzes the generation of Asc (reduced form) from dehydroascorbate (DAsc, oxidized form) in a reaction that requires GSH. Tobacco *DHAR* overexpression lines that have elevated levels of reduced Asc in guard cells show hyposensitivity in stomatal response to ABA and H_2_O_2_ and these plants are drought susceptible. In contrast, DHAR antisense tobacco lines show drought tolerance (Chen and Gallie, 2004). These findings indicate that Asc redox state plays an important regulatory role in ABA and H_2_O_2_ mediated stomatal responses. Altered redox state and stomatal aperture in mutants defective in GSH synthesis are well established (Okuma et al., 2011; Munemasa et al., 2013). Negative regulation of methyl jasmonate (MeJA)-induced stomatal closure by GSH in *A. thaliana* has been demonstrated (Akter et al., 2013). In addition, GSH peroxidases are known to function as redox transducers as well as scavengers in ABA-mediated stress responses (Miao et al., 2006). Thus, understanding redox changes and their regulation and coordination with stomatal functions would provide new insights into guard cell signaling networks.

Stomatal guard cells have a thick cuticular layer containing high concentrations of wax-bound phenolics that provide protection against UV radiation (Karabourniotis et al., 2001; He et al., 2013) and form a constitutive defense barrier against pathogens and insects. Intracellular phenolics and flavonoids synthesized from the phenylpropanoid pathway are also responsible for cellular defense and pigmentation among other functions. Flavonoids protect plants from UV-B irradiation (Li et al., 1993) and also function as stress-induced antioxidants (Dixon and Paiva, 1995). Flavonols accumulate in guard cells of *A. thaliana*, but not in the surrounding pavement cells (Watkins et al., 2014). Enhanced flavonol content and decreased ROS levels upon ethylene (ET) treatment in guard cells were correlated with a reduction in the rate of stomatal closure in response to ABA. The results suggest that flavonols may quench the ABA-dependent ROS burst (Watkins et al., 2014). Moreover, some flavonoids, such as quercetin, apigenin, and kaempferol, have functions similar to synthetic auxin transport inhibitors, so changes in the synthesis or deposition of specific flavonoids within cells may act to change the rate or direction of auxin transport (Winkel-Shirley, 2002). Given that ABA reduces guard cell auxin concentrations (Jin et al., 2013), it would be interesting to further investigate the interrelationships between flavonoids, ROS, ABA, and guard cell auxin transport.

## Role of Lipid Signaling in Stomatal Movement and Development

Lipids are essential for membrane formation and energy storage. In addition, lipids and their metabolites are also important cellular signaling molecules, including in stomatal regulation. For instance, lipid-based secondary messengers that positively regulate guard cell ABA signaling and stomatal closure include phosphatidic acid (PhA), phosphatidyl-inositol-3-phosphate (PI3P), inositol-1,4,5-trisphosphate (IP3), inositol-6-phosphate (IP6), and sphingolipids (Kim et al., 2010; Figure 1).

Phosphoinositides play important roles in guard cell signaling. Phospholipase C (PLC) hydrolyses phosphatidylinositol 4,5-bisphosphate (PIP2) to produce 1,2-diacylglycerol (DAG) and IP3. ABA induced the production of IP3 in *Vicia faba* guard cells (Lee et al., 1996), and cytosolic Ca^2+^ elevation and subsequent stomatal closure occurred upon experimental elevation of cytosolic IP3 in *Commelina communis* guard cells (Gilroy et al., 1990). Increases in guard cell PI3P and PI 4-phosphate (PI4P; the products of PI 3-kinase (PI3K) and PI 4-kinase (PI4K) activities, respectively) induce stomatal closure mediated by ABA-induced ROS generation (Jung et al., 2002; Park et al., 2003). IP6 is generated in guard cells in response to ABA. IP6 is an endomembrane-acting Ca^2+^-release signal that inhibits the inwardly rectifying K^+^ channel, which would then inhibit stomatal opening (Lemtiri-Chlieh et al., 2003).

PhA, a product of phospholipase Dα1 (PLDα1) activity, is a positive regulator in ABA-induced ROS and NO production that promotes stomatal closure (Zhang et al., 2009). In *A. thaliana* guard cells, NO synthesis is positively regulated by both ABA and ROS, and interaction of PhA with the two NADPH oxidases, AtrbohD and AtrbohF (Kwak et al., 2003), is necessary for ABA-induced ROS production (Zhang et al., 2009). The NADPH oxidase-deficient double mutant *atrbohD/F* shows impaired ABA induction of NO production and stomatal closure, indicating that ROS production is necessary for NO production. Application of NO scavengers can inhibit ROS-mediated stomatal closure, indicating that NO is required for ROS-promoted stomatal closure. In contrast, application of NO cannot induce ROS production in *A. thaliana* guard cells (Bright et al., 2006). These findings indicate that PhA functions upstream of ROS production and ROS function upstream of NO production.

The lipid metabolite sphingosine-1-phosphate (S1P) is a product of sphingosine kinase (SPHK) activity, which uses the long-chain amine alcohol sphingosine as a substrate. S1P induced increases of cytosolic (Ca^2+^) (Ng et al., 2001) and stimulated stomatal closure in *C. communis* and *A. thaliana* (Ng et al., 2001, Coursol et al., 2003). The *A. thaliana* genome encodes two functional SPHK genes, *SPHK1* and *SPHK2* (Worrall et al., 2008; Guo et al., 2011). Both SPHKs can use sphingosine and phyto-sphingosine as substrates to produce S1P and phyto-S1P, respectively. Both S1P and phyto-S1P induce stomatal closure in *A. thaliana* (Coursol et al., 2005). S1P inhibits inward K^+^ channels and promotes slow anion channel activity in *A. thaliana* guard cell protoplasts, which in turn cause inhibition of stomatal opening and promotion of stomatal closure, respectively (Coursol et al., 2003). In *A. thaliana*, a functional G-protein α-subunit (GPA1) is required for S1P regulation of ion channels (Coursol et al., 2003). In *A. thaliana*, PhA interacts with SPHKs, promoting substrate binding, which in turn increases SPHK activity. Phyto-S1P induces PhA production in wild type (WT) *A. thaliana*, but not in the *pldα* mutant, indicating a positive regulatory role of phyto-S1P in PLDα-mediated PhA production. It has been suggested that phyto-S1P promotes PLDα activity by increasing cytoplasmic Ca^2+^ concentration (Guo and Wang, 2012). These findings indicate that phyto-S1P and PhA are dependent on each other via positive feedback regulation.

A guard cell-specific and ABA-independent oxylipin pathway was recently reported (Montillet and Hirt, 2013). Derived from complex membrane lipids, unesterified fatty acids are catalyzed by lipoxygenase (LOX) into various oxylipin products, such as JA, fatty acid hydroperoxides, and reactive electrophile species (RES) oxylipins, and these can induce stomatal closure at nanomolar concentrations (Montillet et al., 2013). *A. thaliana lox1* mutants were as sensitive to exogenously applied ABA as WT plants, suggesting that LOX1 activity is not involved in ABA-induced stomatal closure. In addition, a transgenic SA-deficient NahG line, and the two SA biosynthesis mutant lines, *sid1-1* and *sid2-1*, responded normally to ABA, but were non-responsive to RES oxylipins. In addition, *lox1* mutant lines were as sensitive to SA (100 μM) as WT, demonstrating that exogenously applied SA compensated for the *LOX1* deficiency. The results indicate that SA is required to convey the RES oxylipin signal, but not the ABA-mediated signal, leading to stomatal closure.

Naturally occurring saturated short, straight chain fatty acids, such as decanoic and undecanoic acids, can inhibit stomatal opening and cause stomatal closure in epidermal strips of *C. communis* (Willmer et al., 1978). In contrast, some polyunsaturated fatty acids, such as linolenic and arachidonic acid enhance stomatal opening and inhibit stomatal closing, consistent with their promotion of inward K^+^ channel activity and inhibition of outward K^+^ channel activity (Lee et al., 1994). Very-long-chain polyunsaturated fatty acids (VLCPUFAs), such as eicosapentaenoic acid (20:5 δ^5,8,11,14,17^) are abundant lipids in several key plant pathogens (Sun et al., 2013), and may elicit plant defense responses, including stomatal closure. Interestingly, it was shown that exogenous application of eicosadienoic and eicosatrienoic acids to WT plants or endogenous production in the transgenic plants could reduce water loss from excised leaves and confer ABA hypersensitivity to stomatal responses (Yuan et al., 2014). Some fatty acids have been shown to regulate stomatal development, thus affecting the overall plant response to the environment. The *A. thaliana* gene *HIC* (high carbon dioxide) encodes a putative 3-keto acyl coenzyme A synthase (KCS), an enzyme involved in the synthesis of very-long-chain fatty acids (VLCFA) and is a negative regulator of stomatal development in response to CO_2_ (Gray et al., 2000). Mutant *hic* plants exhibit up to a 42% increase in stomatal density in response to a doubling of CO_2_, possibly by preventing the synthesis of component(s) of the extracellular matrix found at the guard cell surface, such as waxes, glycerolipids, sphingolipids, and cutin (Gray et al., 2000). FATTY-ACID DESATURASE4 (FAD4) is required to desaturate palmitic acid (16:0), and the *fad4* mutant is unable to change stomatal index (defined as the percentage of stomata as compared to all the epidermal cells (including stomata) in a unit area of leaf) in response to elevated CO_2_ (Lake et al., 2002). Metabolic profiling of *sdd1* (*STOMATAL DENSITY AND DISTRIBUTION1*) plants, which have three to fourfold higher stomatal density than WT plants, showed a fivefold reduction of unsaturated C16 fatty acids compared to WT, and a concomitant rise in saturated fatty acid 16:0 species (i.e., palmitic acid; Fiehn et al., 2000). The fates of these fatty acids are scarcely known, although it is assumed that some are incorporated into the cutin layers. In *A. thaliana*, mutations of the VLCFA-producing enzymes CER6, CER1, and HIC that are involved in cuticle biosynthesis result in increased stomatal index (Gray et al., 2000). Whether stomatal index/density affects stomatal movement is not clear. Nevertheless, the aforementioned roles of fatty acid metabolites and their metabolic enzymes offer new avenues to elucidate lipid signaling networks in guard cells, which will facilitate engineering of fatty acid metabolism in crops for enhanced stress tolerance and productivity.

## Phytohormone Cross-talk in Stomatal Function

The phytohormone ABA, first reported in plants in the 1960s (Eagles and Wareing, 1963; Ohkuma et al., 1963), is the single most studied metabolite in guard cell physiology owing to its distinct stress (e.g., drought) responsiveness and strong effect on stomatal closure. ABA causes stomatal closure, prevents opening of closed stomata, and reduces transpiration in the leaves of a wide range of species. Stomata accumulate (Cornish and Zeevaart, 1986), catabolize (Grantz et al., 1985), and conjugate exogenously supplied ABA (Grantz et al., 1985; Lee et al., 2006), but to date it is unclear if stomatal opening initially includes or requires depletion of endogenous guard cell ABA (Tallman, 2004). The biosynthesis of ABA from carotenoids in plastids and its catabolism and storage in the cytosol and endoplasmic reticulum in plant cells is well characterized (Nambara and Marion-Poll, 2005). The regulatory network of ABA sensing involve three major components, PYRABACTIN RESISTANCE1 (PYR1)/PYR1-LIKE (PYL)/REGULATORY COMPONENTS OF ABA RECEPTORS (RCAR; i.e., PYR/PYL/RCAR; an ABA receptor; Ma et al., 2009; Park et al., 2009; Joshi-Saha et al., 2011), type 2C protein phosphatase (PP2C; a negative regulator) and SNF1-related protein kinase 2 (SnRK2; a positive regulator), and they offer a double negative regulatory system, (PYR/PYL/RCAR—| PP2C—| SnRK2), which has been well studied (Klingler et al., 2010; Umezawa et al., 2010). PP2Cs inactivate SnRK2s kinases by physical interaction and direct dephosphorylation. Upon ABA binding, PYLs change their conformations and then physically interact and inhibit PP2Cs. However, PYLs inhibit PP2Cs in both the presence and absence of ABA and activate SnRK2s (Zhang et al., 2015). Several natural and artificial compounds interacting with the ABA receptor PYR/PYL/RCAR family are now known (Hitomi et al., 2013). Evolutionary insights obtained from studies on components of the ABA signaling network indicate that PYR/RCAR ABA receptor and ABF-type (ABA-responsive element binding factors) transcription factor families arose during land colonization by plants, while the ABA biosynthesis enzymes have evolved in different plant and fungal specific pathways (Hauser et al., 2011). The structural insights provided from the three-dimensional structures of module PYR/PYL/RCAR-ABA-PP2C pave the way to the design of ABA agonists able to modulate the plant stress response (Santiago et al., 2012).

ABA is transported over short and long distances in plants. Plasma membrane-localized ABA transporters belonging to ATP-binding cassette (ABC; Kang et al., 2010) and nitrate transporter 1/peptide transporter (NRT1/PTR) families are established (Boursiac et al., 2013) and ABA-perception sites were visualized on the plasma membrane of stomatal guard cells (Yamazaki et al., 2003), in addition to internal sites of perception. For instance, application of ABA into the cytosol of *V. faba* guard-cell protoplasts via patch-clamp techniques inhibited inward K^+^ currents thus inhibiting stomatal opening (Schwartz et al., 1994). Although ABA synthesis in guard cells and vascular tissues has been shown (Seo and Koshiba, 2011; Bauer et al., 2013; Boursiac et al., 2013), the relative extent to which guard cells and vascular tissues contribute to the ABA dynamics in guard cells is a topic of ongoing interest. For instance, the recent design, engineering and use of ABAleons with ABA affinities in the range of 100–600 nM to map ABA concentration changes in plant tissues with spatial and temporal resolution in distinct cell types, and in response to low humidity and NaCl in guard cells (Waadt et al., 2014) has promising future applications.

ABA causes alkalization of the guard cell cytosol (Blatt and Armstrong, 1993), which directly enhances outward K^+^ channel activity (Blatt and Armstrong, 1993; Ilan et al., 1994; Miedema and Assmann, 1996), and a sustained efflux of both anions and K^+^ from guard cells contributes to loss of guard cell turgor, thus facilitating stomatal closing. In addition, ABA-induced stomatal closing can be Ca^2+^-dependent or -independent (Schroeder et al., 2001). ABA mediated inhibition of stomatal opening is a process distinct from ABA-induced stomatal closure, and it is unclear if H_2_O_2_ and NO are involved in the ABA inhibition of stomatal opening (Desikan et al., 2004). Even after half a century of research, the role of ABA in guard cell signaling continues to be elucidated (Kim et al., 2010; Yu and Assmann, 2014). ABA content can be decreased via catabolism to phaseic acid (PA), sequestration in the form of an ABA-glucose ester (ABA-GE), which is thought to be physiologically inactive, or deposition in vacuoles (Nambara and Marion-Poll, 2005). Studies on sugar-response mutants indicate that ABA and sugar-response pathways overlap extensively (León and Sheen, 2003). It is known that the sugar sensing effects mediated by HXK are dependent on production of and signaling by ABA (Rolland et al., 2006; Rognoni et al., 2007; Ramon et al., 2008); for example, these interactions take place in mesophyll cells where sugar and HXK inhibit expression of photosynthesis genes (Rolland et al., 2006). Recently, it has been shown that sugar and HXK stimulate the ABA signaling pathway within guard cells, promoting stomatal closure (Kelly et al., 2013). These effects were also observed in epidermal peels, suggesting that sugar and HXK stimulate production of ABA, release of biologically active ABA from inactive ABA pools, and/or inhibition of ABA degradation within guard cells (Koiwai et al., 2004; Christmann et al., 2005; Melhorn et al., 2008; Wasilewska et al., 2008; Zhu et al., 2011). These observations also imply that ABA is probably essential for daily regulation of stomatal aperture even in the absence of water stress (Kelly et al., 2013).

A comprehensive and comparative metabolomics study undertaken in guard and mesophyll cells of *A. thaliana* revealed that following ABA treatment, metabolites are clustered into different temporal modules in guard cells and mesophyll cells (Jin et al., 2013). Guard cell modules differ in WT plants as compared to the modules in the heterotrimeric G-protein α subunit null mutant (*gpa1*), with fewer metabolites showing ABA-altered profiles in *gpa1*, consistent with hyposensitivity of *gpa1* K^+^, anion, and Ca^2+^ channels to ABA (Wang et al., 2001; Fan et al., 2008; Zhang et al., 2011). For instance, the Ca^2+^-mobilizing metabolites S1P and cyclic adenosine 5′-diphosphoribose (cADPR) exhibited weaker ABA-stimulated increases in *gpa1* than in WT guard cells. Phytohormones such as ABA catabolites, i.e., ABA glucose-ester, PA, and dihydrophaseic acid (DiHPA), and indole-3-acetic acid (IAA), JA, MeJA, and methyl salicylate were responsive to ABA, with greater responsiveness in WT than in the *gpa1* guard cells. In particular, IAA concentrations in guard cells declined following ABA treatment in WT guard cells but not in *gpa1* guard cells. These findings are consistent with the observation that exogenous application of IAA activates the guard cell H^+^-ATPase and impairs ABA-inhibition of stomatal opening, and suggest that endogenous ABA in guard cells functions upstream to regulate other endogenous hormones, particularly IAA, consistent with G proteins modulating multiple hormonal signaling pathways. Most phytohormones also showed differential ABA responses in guard cells as compared to mesophyll cells (Jin et al., 2013). In support of the idea that multiple hormones regulate guard cell responses, in *V. faba*, cytokinin and auxin induced stomatal opening (Levitt et al., 1987; Song et al., 2006) in conjunction with decreased H_2_O_2_ production (Song et al., 2006). Salicylic acid (SA) is a ubiquitous phenolic phytohormone involved in stomatal movement. Addition of 1 mM SA to fully opened stomata resulted in a significant reduction (75%) in stomatal aperture (Lee, 1998) in *C. communis*. SA is known to induce stomatal closure accompanied by extracellular ROS production, intracellular ROS accumulation and inward K^+^ channel inactivation (Khokon et al., 2011a). Although both ABA and SA were reported to be needed for stomatal closure in response to pathogens, with SA action upstream of ABA (Melotto et al., 2006), a recent study using the ABA biosynthesis mutant *aba2* and a mutant of JA biosynthesis reported no differences in SA induced stomatal closure in the mutants as compared to WT. The authors concluded that neither ABA nor JA is involved in SA, yeast elicitor, or chitosan-induced stomatal closure in *A. thaliana* (Issak et al., 2013). These results appear to indicate the presence of an ABA independent SA signaling pathway in guard cells, but more research is need to fully resolve the contradictory conclusions in the literature.

In *V. faba* (Zhang et al., 2001) and *Pisum sativum* (Suhita et al., 2004), ABA-mediated stomatal closure is preceded by cytoplasmic alkalization and H_2_O_2_ production, events that also occur during MeJA-mediated stomatal closure. In fact, ABA and MeJA-mediated stomatal closure share several characteristic signaling components, such as Ca^2+^ involvement, protein phosphorylation, cytoplasmic alkalization, ROS production, and modulation of plasma membrane K^+^ channels in the guard cells (Suhita et al., 2004). Extremely low levels of the phytotoxin coronatine (COR), secreted by virulent strains of *Pseudomonas syringae* p.v. *tomato* (*Pst*) act as a JA mimic, activate the JA signaling pathway, and enable the strains to reopen stomata, thereby circumventing host stomatal defense (Montillet and Hirt, 2013). However, unlike COR, exogenous MeJA does not appear to antagonize ABA-induced stomatal closure (Melotto et al., 2006). In fact, the ability of MeJA to regulate stomatal apertures remains controversial (Montillet et al., 2013). Allene oxide synthase (AOS) is a key enzyme in the oxylipin pathway and plays a vital role in production of 12-oxo-phytodienoic acid (12-OPDA, a JA precursor) and JA. Recently, it has been proposed that 12-OPDA, rather than MeJA, acts in promotion of stomatal closure (Savchenko et al., 2014).

The role of brassinosteroids (BRs) in stomatal movements is less established. Brassinolide (BL), the most bioactive BR form, has been shown to promote stomatal closure in *V. faba* (Haubrick et al., 2006), where BL-induced stomatal opening was not observed. Interestingly, low concentrations of epibrassinolide (eBL) promoted stomatal opening in epidermal peels of *Solanum lycopersicum* in the dark, whereas high concentrations of eBL promoted stomatal closure in the light (Xia et al., 2014). Exogenous (apoplastic) and endogenous (cytosolic) BR may act differently, and guard cells of different species may respond differently to BL application. In *S. lycopersicum*, transient H_2_O_2_ production was deemed essential for poising the cellular redox status, which played an important role in BR-induced stomatal opening (Xia et al., 2014). BR promoted stomatal closure through apparent biosynthesis of ABA, while stomatal opening was dependent on the GSH redox status of the guard cells. It was proposed that GSH regeneration and/or biosynthesis, leading to a reduced redox status, strictly controls the ROS level and negatively regulates the ABA response pathway, and that BR can directly induce ROS production independently of ABA via NADPH oxidase.

ET and its precursor 1-aminocyclopropane-1-carboxylic acid (ACC) activate the production of H_2_O_2_ in guard cells and induce stomatal closure in *V. faba* (Song et al., 2014), and the closure was preceded by elevated ROS generated by NADPH oxidases (Desikan et al., 2006). However, the ET effect varies depending on species and conditions. For example, ET promotes stomatal closure in *Arachis hypogaea* (Pallas and Kays, 1982) and *A. thaliana* (using intact leaves; Desikan et al., 2006), but evokes stomatal opening in *Dianthus caryophyllus* and *S. lycopersicum* (Madhavan et al., 1983), *V. faba* (Levitt et al., 1987), and *A. thaliana* (using epidermal peels; Tanaka et al., 2005). The ET effect on stomatal opening was attributed to its impairment of ABA regulation of stomatal closure (Tanaka et al., 2005), but recently, it was shown in *A. thaliana* that ET mediated BR-induced stomatal closure via Gα protein-activated AtrbohF-dependent H_2_O_2_ production and subsequent Nia1-catalyzed NO production (Ge et al., 2015; Shi et al., 2015). Nonetheless, the exact mechanisms underlying the different ET effects are unknown.

## Nitrogen and Sulfur Rich Metabolites in Guard Cell Signaling

Nitrogenous bases in the form of purines and pyrimidines form an essential pool of nitrogen in plant cells. Nitrogenous metabolites have been extensively studied as metabolic intermediates and signaling molecules in stomatal movement and guard cell function. Important nitrogenous signaling molecules, such as cADPR, a metabolite derived from nicotinamide adenine dinucleotide (NAD; Wu et al., 1997), play important roles in guard cell ABA signaling. Injection of cADPR into guard cells resulted in [(Ca^2+^)_cyt_] increases and turgor reduction. When guard cells were preloaded with the cADPR antagonist 8NH_2_-cADPR, a slowing of stomatal closure was observed in response to ABA (Leckie et al., 1998). Recently, it was established that inhibition of the poly (ADP-R) polymerase activity correlated with increased number of stomata in *A. thaliana* leaves (Schulz et al., 2014), highlighting the role of poly (ADP-R) metabolism in stomatal development. Another nucleotide-related metabolite, cyclic guanosine monophosphate (cGMP), has been implicated in ABA-induced stomatal closure by acting downstream of H_2_O_2_ and NO in the signaling pathway by which ABA induces stomatal closure (Dubovskaya et al., 2011). H_2_O_2_ and NO-induced cytosolic calcium increases [(Ca^2+^)_cyt_] were cGMP-dependent, positioning cGMP upstream of (Ca^2+^)_cyt_, and involved the action of the type 2C protein phosphatase, ABI1. Increases in cGMP were mediated through the stimulation of guanylyl cyclase by H_2_O_2_ and NO (Dubovskaya et al., 2011). The nitrated form of cGMP (8-nitro-cGMP) is a positive regulator in promotion of stomatal closure (Joudoi et al., 2013). NO and cGMP induce the synthesis of 8-nitro-cGMP in guard cells in the presence of ROS leading to the hypothesis that NO-dependent guanine nitration of cGMP may occur in plants and the resulting 8-nitro-cGMP acts as a signaling molecule that activates cADPR production in guard cells. By contrast, a positive role for cGMP in kinetin- and natriuretic peptide–induced stomatal opening in *Tradescantia albiflora* (Pharmawati et al., 1998) and in auxin-induced stomatal opening in *C. communis* and *A. thaliana* (Cousson and Vavasseur, 1998; Cousson, 2003) also has been recognized. Furthermore, application of 8-bromo-cGMP, a membrane-permeant cGMP analog, causes stomata to open in the dark (Cousson, 2003; Joudoi et al., 2013), but 8-nitro-cGMP does not. These results lead to the conclusion that cGMP and its nitrated derivative play different roles in guard cell signaling, wherein cGMP promotes stomatal opening in the dark, while 8-nitro-cGMP promotes stomatal closure in the light (Joudoi et al., 2013).

Another important group of N-containing specialized metabolites in plants are polyamines. Exogenous application of polyamines, such as 1 mM spermine, inhibit stomatal opening by inhibiting inwardly rectifying K^+^ channels (Liu et al., 2000). Application of spermidine also promotes stomatal closure but the mechanism is unknown as outward K^+^ channels and anion channels are not affected (Liu et al., 2000). On the other hand, (acetyl-)1,3-diaminopropane (DAP), a product of oxidative deamination of spermidine and spermine, suppresses anionic currents, and increases those of inwardly rectifying K^+^ channels, and may induce membrane hyperpolarization and extracellular acidification by activating the H^+^ ATPase, thus restraining stomatal closing (Jammes et al., 2014). These mechanisms act antagonistically to ABA. It is thought that during acclimation to low soil-water availability, acetyl-DAP prevents complete stomatal closure (Jammes et al., 2014). Moreover, DAP and such amine oxidase reaction products are precursors of γ-amino butyric acid, alkaloids, β-alanine, and other uncommon polyamines that play significant roles in stress tolerance and defense (Bouchereau et al., 1999). In fact, based on proteome analysis in *Brassica napus* guard cells, ABA-responsive proteins that decrease in abundance include those involved in spermidine synthesis, purine metabolism, and alkaloid biosynthesis pathways (Zhu et al., 2010).

Glucosinolates are N- and S-containing specialized metabolites in plants that have been shown to be present in guard cells. Glucosinolate-myrosinase systems in Brassicales, especially *A. thaliana*, are well understood in plant-herbivore interactions and defense against pathogens (Yan and Chen, 2007; Andersson et al., 2015). However, recent evidence from proteomic investigations has indicated that glucosinolates are required for ABA responses of guard cells (Zhao et al., 2008, Zhu et al., 2010, 2014). THIOGLUCOSIDE GLUCOHYDROLASE1 (TGG1), encoding a myrosinase that catalyzes the production of isothiocyanates (ITC) from glucosinolates, is highly abundant in guard cell proteomes. In fact, myrosinases are proposed to redundantly function downstream of ROS production and upstream of cytosolic Ca^2+^ elevation in ABA and MeJA signaling in guard cells (Islam et al., 2009). *A. thaliana tgg1* mutants are hyposensitive to ABA inhibition of guard cell inward K^+^ channels and stomatal opening. In addition, thiol-reagents such as ITCs have been shown to be potent inducers of stomatal closure, possibly via covalent reactions with RES oxylipin targets (Montillet et al., 2013). Some of the glucosinolate-producing plant species, such as *Brassica juncea,* produce 2-propenylglucosinlate, which can be hydrolyzed to allylisothiocyanate (allylITC). Exogenous application of allyl-ITC was found to induce stomatal closure (Khokon et al., 2011b). The stomatal closure by allylITC was induced via production of ROS and NO, and elevation of cytosolic Ca^2+^. In addition, other ITCs, nitriles, and thiocyanates (e.g., 3-butenenitrile and ethyl thiocyanate) have also been shown to induce foliar ROS generation and stomatal closure (Hossain et al., 2013). Manipulation of glucosinolate metabolic pathways by plant metabolic engineering and breeding approaches may lead to development of crop varieties with combined disease and drought resistance. Recently, another sulfur containing compound, hydrogen sulfide (H_2_S), generated by L-cysteine desulfhydrase was shown to act upstream of NO to modulate ABA-dependent stomatal closure (Scuffi et al., 2014).

## Conclusion

The plant leaf metabolome can boast as many as 5,000 different metabolites (Bino et al., 2004). Considering the roles of established metabolites in guard cell functions, we have begun the heydays of functional genomics, fluxomics, and systems biology toward understanding of this highly sophisticated single cell type model system. Although studies on guard cell metabolism are highly biased toward ABA and osmolytes owing to their primary importance in stomatal movement, the identification of additional critical metabolites (as shown in Figure 1) underlying or correlated with stomatal movement will form a solid foundation toward a broader understanding of optimal plant adaptation to environmental changes. For example, although progress in the study of stomatal movement in plant immunity has been made (Zhang et al., 2008), a deeper mechanistic understanding is required to harness the potential for generation of disease resistant crops. Information currently available has revealed universal and diverse metabolites and pathways leading to stomatal responses.

Many years of traditional breeding has unknowingly selected varieties with cool leaf temperature in some species, i.e., larger stomatal opening for higher yield. For instance, in Pima cotton (*Gossypium barbadense* L.) and bread wheat, increased stomatal conductance led to lint and grain yield increases respectively (Lu et al., 1998). Furthermore, in cotton, stomatal conductance and leaf cooling were significantly correlated with fruiting prolificacy and yield during the hottest period of the year (Radin et al., 1994). Understanding the functions and molecular networks of the regulatory metabolites of stomatal functions would open avenues for development of “smart” crops, providing a unique platform for endeavors at the genetic level to favor food security and human nutrition. Although we did not focus on the roles of stomatal ontogeny, shape, size, and distribution, which can also significantly affect plant water balance, growth and biomass, the engineering of stomatal development and response as a means to improve water use efficiency is an attractive approach to improve drought tolerance in crops (Schroeder et al., 2001). Guard cell metabolomics and systems biology hold the potential to unravel key molecular networks that control plant productivity and defense in a changing climate.

### Conflict of Interest Statement

The authors declare that the research was conducted in the absence of any commercial or financial relationships that could be construed as a potential conflict of interest.
